# Cerebellar infarction as the initial presentation of IgG4‐related disease

**DOI:** 10.1002/ccr3.5614

**Published:** 2022-03-17

**Authors:** Branden Ireifej, Umaima Dhamrah, David Song, Joyce Bitar, Vikash Jaiswal, Gaurav Nepal, Nibesh Pathak, Majd Freijat

**Affiliations:** ^1^ Department of Internal Medicine Icahn School of Medicine at Mount Sinai Elmhurst Hospital Center New York City New York USA; ^2^ 25063 Metropolitan Hospital Center New York City New York USA; ^3^ Larkin Community Hospital South Miami Florida USA; ^4^ Department of Internal Medicine Tribhuvan University Institute of Medicine Kathmandu Nepal; ^5^ Flow Rheumatology Scottsdale Arizona USA

**Keywords:** cerebellum, CNS, IgG4‐RD, lymphoplasmacytic, plasma cells

## Abstract

Although IgG4‐RD has CNS manifestations, cerebellar involvement has only been reported in three cases. Our patient presented with cerebellar symptoms, several cerebellar infarcts were evident on the brain MRI, and CT abdomen revealed retroperitoneal tumor. Endoscopic biopsy confirmed IgG4‐RD. Steroids are the first‐line therapy for IgG4‐RD, but our patient was lost to follow‐up before treatment.

## Graphical abstract

IgG4‐related disease (IgG4‐RD) is a multiorgan, inflammatory disease with specific histopathological features. This is one of the few cases in which a patient presented with cerebellar symptoms as the initial symptoms for IgG4‐RD.

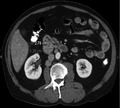

## 1 INTRODUCTION

IgG4‐RD is a systemic fibroinflammatory disease characterized by significant infiltration of IgG4‐positive plasma cells in afflicted tissues, with or without elevated IgG4 plasma levels. Many essential pathological and clinical similarities exist in affected organs, including tumor‐like enlargement, lymphoplasmacytic infiltration rich in IgG4‐positive plasma cells, and varying degrees of fibrosis with a characteristic "storiform" pattern.^1^ IgG4‐RD was first described in 2003 for disorders that were previously thought to be unrelated—for example, autoimmune pancreatitis type I, chronic sclerosing cholangitis, retroperitoneal fibrosis, hypertrophic pachymeningitis, and Riedel's thyroiditis, among others—but were found to co‐occur in some patients with similar histological findings.^2^ IgG4‐RD has been identified with increasing frequency since then. However, knowledge of diagnostic and therapeutic methods for the management of individuals with IgG4‐RD is currently limited to tertiary care facilities, and the illness is still misdiagnosed as neoplastic, inflammatory, or infectious disorders. IgG4‐RD symptoms vary greatly depending on the organ affected.^2^, ^3^ The salivary and lacrimal glands, pancreas and biliary tract, and kidneys are the most usually afflicted organs; however, any organ can be implicated, and multiorgan illness can occur. Patients with IgG4‐RD have been documented to develop hypertrophic pachymeningitis, hypophysitis, or an inflammatory pseudotumor in the central nervous system.^4^ However, to the best of our knowledge, this is the first few cases of IgG4‐RD with cerebellar symptoms caused by underlying cerebellar infarcts.

## 2 CASE REPORT

A 60‐year‐old man with a history of hyperlipidemia presented to the emergency department with worsening dizziness and blurry vision. He has had these symptoms for several months prior to arrival. On further examination, he also endorsed pain in his right hand that started around several months prior; the pain was described as numb and uncomfortable. He also noted some discoloration of his right fingers. Review of systems was negative for fevers, chills, shortness of breath, chest pain, abdominal pain, rash, joint pain, or family history of autoimmune disease. He is originally from Mexico and denied any use of alcohol, cigarettes, or illicit drugs. Vital signs were within normal limits. Physical examination was notable for a faint, purple discoloration of the tip of the right fingers. The patient was admitted for further management.

**FIGURE 1:**
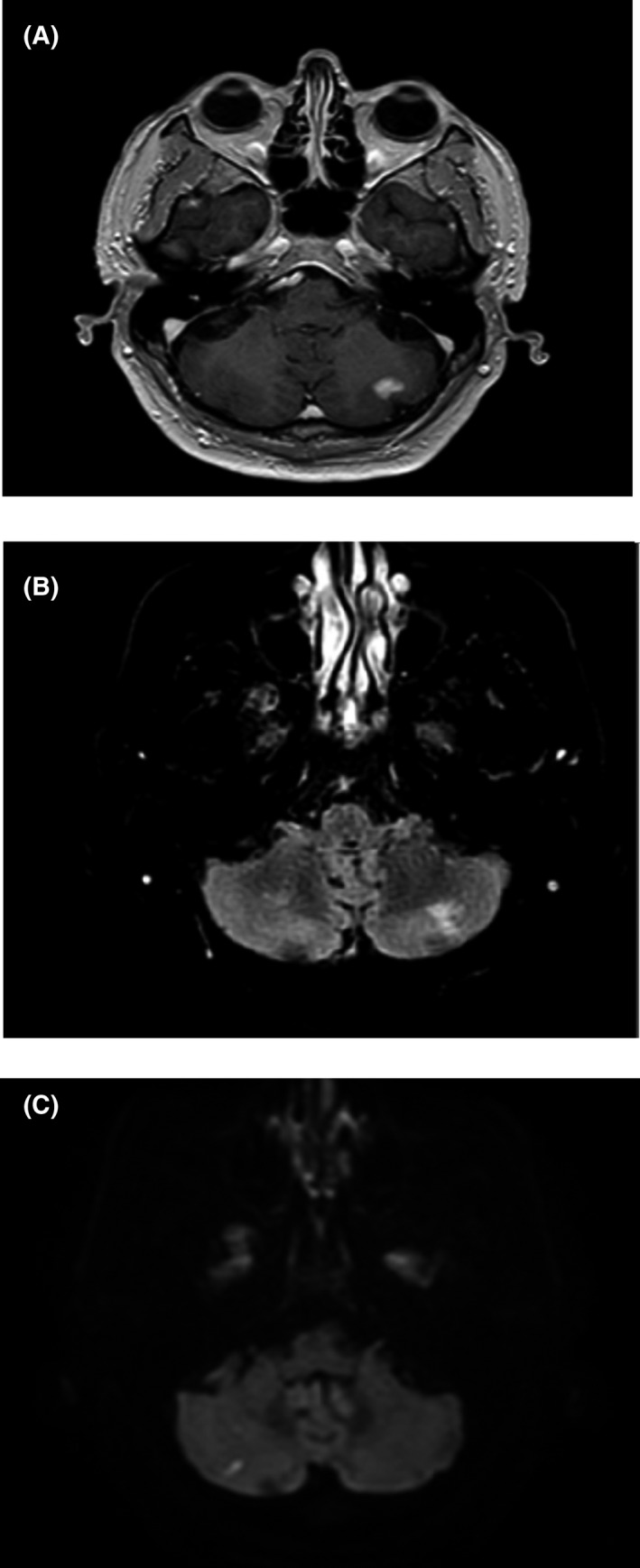
Magnetic resonance (MR) imaging of the brain revealed a small, acute right cerebellar infarct, subacute left cerebellar infarct, and mild bifrontal chronic microvascular ischemic changes (A = T1 sequence, B = T2 FLAIR sequence, and C = DWI sequence)

**FIGURE 2:**
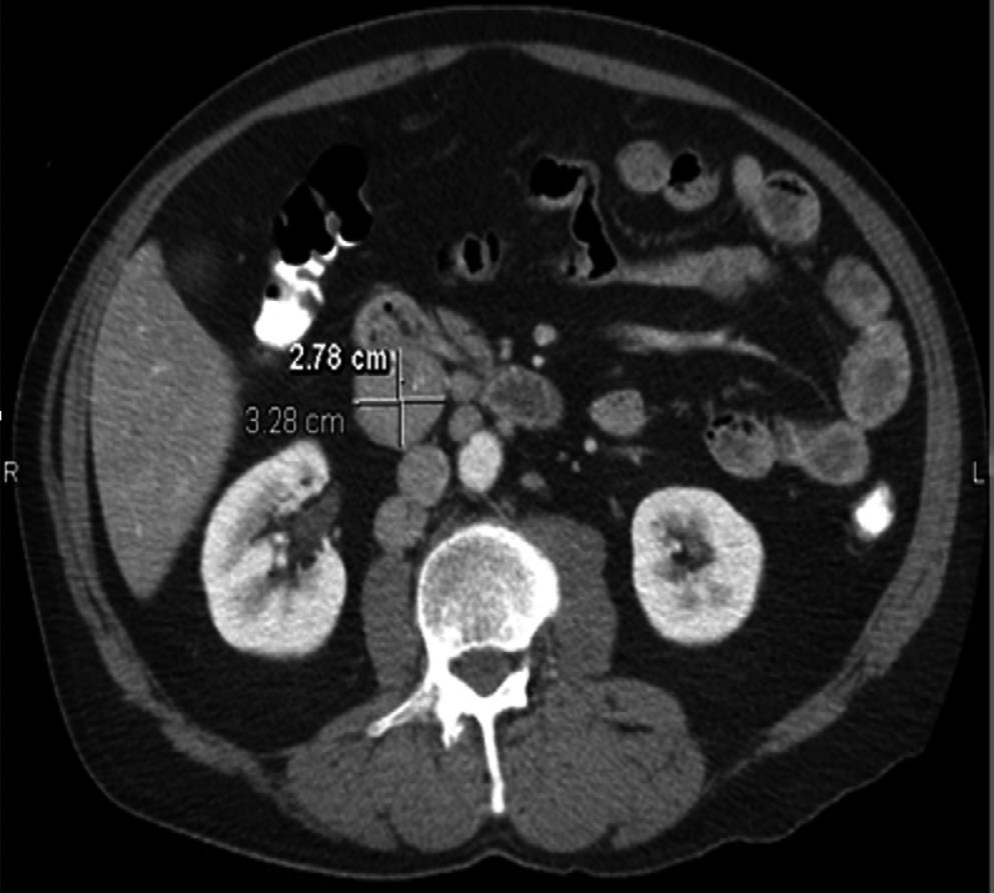
Computed tomography of abdomen showed a 2.8 × 3.3 × 3.5 solid partially calcified right retroperitoneal mass arising from the pancreatic head/duodenum and associated mild retroperitoneal lymphadenopathy

Initial laboratory work showed an elevated erythrocyte sedimentation rate of 48 mm/h and C‐reactive protein of 13.9 mg/L. His basic metabolic panel, complete blood count, hemoglobin A1C, antinuclear antibody, cryoglobulin, rheumatoid factor, antiphospholipid serology, complement 3 and 4, and hepatitis panel were all unremarkable. Magnetic resonance (MR) imaging of the brain with and without contrast revealed a small, acute right cerebellar infarct, subacute left cerebellar infarct, and mild bifrontal chronic microvascular ischemic changes (Figure 1). MR angiography of head and neck with contrast was unremarkable. Orbital MR imaging was performed due to his blurry vision, which revealed no abnormalities. Echocardiogram showed a normal ejection fraction with no other abnormalities. Computed tomography of the chest, abdomen, and pelvis showed a 2.8 × 3.3 × 3.5 solid partially calcified right retroperitoneal mass arising from the pancreatic head/duodenum (Figure 2). Associated mild retroperitoneal lymphadenopathy was noted with no pancreatic ductal dilatation.

The gastroenterology team was consulted for an endoscopic ultrasound with biopsy. The results showed lymph node tissue with plasmacytosis and >40% plasma cells positive for IgG4, compatible with IgG4 lymphadenopathy. The specimen was composed of lymphoid tissue lacking germinal centers with plasmacytosis. Immunohistochemical staining was performed. Cytokeratin AE1/AE3 was negative for epithelial malignancy. CD20 demonstrated an increase in B cells, and CD138 showed an increase in plasma cells. Kappa and lambda in situ hybridization showed polytypic expression. CD3 and CD43 highlighted background T cells. HHV8 was also negative, providing no support for Castleman disease.

Prior to the pathology results, the patient was scheduled to be seen by hematology/oncology service and was sent home on apixaban, clopidogrel, and aspirin. Steroids were not initiated given the etiology of the stroke was believed to stem from his risk factors (age, hyperlipidemia, and possible pancreatic cancer). However, the patient was discharged and lost to follow‐up before being aware of the pathology results.

## 3 DISCUSSION

IgG4‐RD is a multiorgan, fibroinflammatory condition with tumefactive lesions of unknown etiology and characteristic histopathological features. Virtually, any organ can be involved, most commonly being the pancreas, kidneys, orbital adnexal structures, and retroperitoneum. The pathological hallmark of this disease is dense lymphoplasmacytic infiltrate with IgG4‐positive plasma cells.^5^ The pathophysiology is not well understood, but it has been proposed that the effectiveness of B‐cell depletion therapy in IgG4‐RD suggests that B lymphocytes and other cells of this lineage play an important pathological role.^6^ The presentation of this disease is subacute due to enlargement of organs and can present in a multitude of ways generally in men of the sixth decade. Diagnosing IgG4‐RD is based on both clinical and histopathological features coupled with laboratory testing, with several criteria scoring options. However, histopathology is the current "gold standard" for diagnosis. Primary histopathology seen is lymphoplasmacytic inflammation (>40% plasma cells positive for IgG4) dense with lymphoid aggregate and germinal center formation.^7^, ^8^

Based on the Japanese comprehensive clinical diagnostic criteria for IgG4‐RD, our patients’ presenting symptoms were likely due to IgG4‐related disease.^3^ Our patient presented primarily with cerebellar symptoms, which is an unusual presentation for this disease. Neurologic manifestations for the disease generally present as hypertrophic pachymeningitis, hypophysitis, or an inflammatory pseudotumor.^3^ The patient was treated to have multiple cerebellar infarcts on MR imaging of the brain. On abdominal imaging, a right retroperitoneal mass arising from the pancreatic head/duodenum was found. Subsequent biopsy showed >40% plasma cells positive for IgG4, diagnostic of IgG4‐RD.

The patient's multiple cerebellar infarcts and discoloration of the fingertips could indicate a component of an underlying vasculitis. There have been only a small number of cases reporting cerebral vasculitis associated with IgG4‐RD, but none have reported cerebellar symptoms prior to diagnosis of IgG4‐RD. Cerebral vasculitis due to IgG4‐RD reported with pathological findings was documented in three previous cases (Table 1). In the first case, the patient presented with nystagmus and respiratory failure. Imaging demonstrated dolichoectasia of the vertebral basilar artery.^9^ The second was a case presenting as progressive dementia and spastic hemiparesis. In this case, pathological findings of the brain biopsy revealed parenchyma involvement with lymphocyte infiltration.^10^ The third case had no presenting symptoms but had hyperintense lesions in the right middle temporal gyrus, leptomeningeal enhancement on imaging in a patient with biopsy proven IgG4‐RD.^4^ In the latter, corticosteroids resulted in an asymptomatic stage. However, our patient was discharged and lost to follow‐up prior to trial of corticosteroids. This is the major limitation of our case report.

**TABLE 1:** Reported cases of cerebral vasculitis due to IgG4‐RD

Author	Clinical presentation	Imaging/histopathology
Toyoshima et al.	Nystagmus and respiratory failure	Dolichoectasia of the vertebral basilar artery
Usami et al.	Progressive dementia and spastic hemiparesis	Parenchyma involvement with lymphocyte infiltration
Regev et al.	Asymptomatic	Leptomeningeal enhancement with hyper‐intense lesions in the right middle temporal gyrus
Our case	Worsening dizziness and blurry vision	Multiple cerebellar infarcts

In conclusion, cerebellar symptoms in IgG4‐RD are an atypical initial presentation that has been reported in only a handful of cases. Based on previous reports, early initiation of corticosteroids can result in substantial improvement. While this disease is not entirely understood, it is important for clinicians to be aware of this unique presentation and to have a high clinical index of suspicion when presented.

## CONFLICT OF INTEREST

The authors declare that they have no conflicts of interest to declare.

## AUTHORS CONTRIBUTIONS

BI, UD, DS, and MF were involved in patient care (diagnosis, treatment, and follow‐up). VJ, GN, and NP contributed to the writing of the manuscript, editing, and manuscript revision. All authors approved the final version.

## ETHICAL APPROVAL

Ethical approval is not required for this type of study in accordance with local guideline.

## CONSENT

Written informed consent was obtained from the patient for publication of this case report.

## Data Availability

All data generated or analyzed during this study are included in this article.
